# Non-insulin-based insulin resistance indices for predicting all-cause mortality and renal outcomes in patients with stage 1–4 chronic kidney disease: another paradox

**DOI:** 10.3389/fnut.2023.1136284

**Published:** 2023-05-15

**Authors:** Feng-Ching Shen, Hugo You-Hsien Lin, Wei-Chung Tsai, I-Ching Kuo, Yi-Kong Chen, Yu-Lin Chao, Sheng-Wen Niu, Chi-Chih Hung, Jer-Ming Chang

**Affiliations:** ^1^Division of Nephrology, Department of Internal Medicine, Kaohsiung Medical University Hospital, Kaohsiung Medical University, Kaohsiung, Taiwan; ^2^Department of Internal Medicine, Kaohsiung Medical University Hospital, Kaohsiung Medical University, Kaohsiung, Taiwan; ^3^Department of Internal Medicine, Kaohsiung Municipal Ta-Tung Hospital, Kaohsiung, Taiwan; ^4^Faculty of Medicine, College of Medicine, Kaohsiung Medical University, Kaohsiung, Taiwan; ^5^Division of Cardiology, Department of Internal Medicine, Kaohsiung Medical University Hospital, Kaohsiung Medical University, Kaohsiung, Taiwan; ^6^Graduate Institute of Clinical Medicine, College of Medicine, Kaohsiung Medical University, Kaohsiung, Taiwan

**Keywords:** non-insulin-based insulin resistance indices, TyG index, stage 1–4 chronic kidney disease, obesity paradox, all-cause mortality, renal outcome

## Abstract

Non-insulin-based insulin resistance (IR) indices serve as the indicators of metabolic syndrome (MetS) but have limited value for predicting clinical outcomes. Whether the obesity paradox affects the predictive value of these indicators in patients with chronic kidney disease (CKD) remains unknown. We investigated whether MetS and non-insulin-based IR indices can predict all-cause mortality and renal outcomes in a prospective observational study with stage 1–4 CKD Asians (*N* = 2,457). These IR indices were associated with MetS. A Cox regression model including body mass index (BMI) revealed an association between MetS and renal outcomes. Among the IR indices, only high triglyceride–glucose (TyG) index was associated with adverse renal outcomes: the hazard ratio of Q4 quartile of the TyG index was 1.38 (1.12–1.70). All-cause mortality was marginally associated with MetS but not high IR indices. Low TyG and TyG–BMI indices as well as low BMI and triglyceride were paradoxically associated with increased risks of clinical outcomes. The triglyceride-to-high-density lipoprotein cholesterol ratio and metabolic score for IR indices were not associated with clinical outcomes. In conclusion, MetS and TyG index predict renal outcome and obesity paradox affects the prediction of IR indices in patients with stage 1–4 CKD.

## 1. Introduction

A continual increase has been noted in the prevalence of metabolic syndrome (MetS); MetS was estimated to affect approximately one-quarter of the world’s population in 2018 (1). MetS is defined as elevated blood pressure, dyslipidemia, increased fasting blood glucose levels, and central obesity (2). MetS is independently associated with the risks of chronic kidney disease (CKD) (3, 4) and microalbuminuria (4). In patients with CKD, the prevalence of MetS has been reported to be approximately 65% (5). Furthermore, MetS is strongly associated with all-cause mortality (6) and adverse renal outcomes (7). However, a study involving 25,868 patients with stage 3 or 4 CKD (proportion of Caucasian patients, 86.9%) revealed an association of MetS with progression to end-stage renal disease (ESRD) but not with all-cause mortality (8). A Taiwanese study suggested that the effects of MetS on CKD progression are prominent only in patients with early-stage CKD without diabetes (9). We previously reported a U-shaped association between waist-to-hip ratio and all-cause mortality in patients with CKD (10). Further studies are required to identify the associations between MetS, CKD progression, and all-cause mortality in patients with CKD.

Insulin resistance (IR) is a key indicator of MetS (11, 12). IR is common in patients with CKD, which contributes to renal function deterioration and increased cardiovascular disease risk (13). The mechanism of IR involves the common pathways of metabolite-driven gluconeogenesis and ectopic lipid accumulation (14). In their study involving a Swedish cohort (*n* = 8,980), Wagner et al. revealed that individuals with IR had a high risk of diabetic nephropathy than general population (15). The gold standard for measuring IR is the hyperglycemic clamp technique, which helps quantify the sensitivity of beta cells to glucose and that of tissues to insulin (16). Alternative methods of IR measurement include insulin-based approaches, such as homeostasis model assessment of IR (HOMA-IR) (17) and the quantitative insulin sensitivity check index (18), and non-insulin-based approaches, such as the triglyceride (TG)–glucose (TyG) index (19), TyG–body mass index (BMI) (20), TG-to-high-density lipoprotein cholesterol (HDL-c) ratio (21), and metabolic score for IR (METS-IR) (22). Non-insulin-based approaches predict IR by substituting insulin assessments with assessments of fasting TG level, glucose level, lipoprotein level, or BMI; these surrogate indices are easily accessible in clinical practice.

Non-insulin-based IR indices are strongly associated with MetS. The TyG index has been widely used as an indicator of IR (23); its efficacy may be higher than that of HOMA-IR (24). Furthermore, the TyG–BMI index (20), TG/HDL-c ratio (25), and METS-IR (22) help predict MetS (26, 27). However, few studies have focused on the value of these indices for predicting clinical outcomes in patients with CKD. A high TyG index is strongly associated with renal function progression (28, 29) and diabetic nephropathy in patients with type 2 diabetes mellitus (DM) (30, 31). This index is also associated with all-cause mortality and cardiovascular death (32–34). We previously revealed reverse associations between BMI, all-cause mortality (10), and renal outcomes (35)—termed the obesity paradox—in patients with CKD. Whether clinical outcomes predicted using non-insulin-based IR indices are also affected by the obesity paradox remains unknown.

Considering the obesity paradox, we investigated whether MetS and non-insulin-based IR indices can predict all-cause mortality and renal outcome in patients with stage 1–4 CKD.

## 2. Materials and methods

### 2.1. Study design and participants

This prospective observational study, the Integrated CKD Care Program in Kaohsiung for Delaying Dialysis, involved two affiliated hospitals of Kaohsiung Medical University, southern Taiwan, and was conducted between November 11, 2002, and May 31, 2009 (36). We extended the follow-up period to 31 December 2014. The present study included patients with stage 1–4 CKD who did not receive renal replacement therapy. Patients with acute kidney injury, defined as a >50% decrease in estimated glomerular filtration rate (eGFR; calculated using the modification of diet in renal disease equation) within 3 months; patients who were lost to follow-up within 3 months; and patients with CKD were excluded from this study. Finally, this study included 2,457 patients with stage 1–4 CKD and a BMI of 15.0–35.0 kg/m^2^. To investigate the association of non-insulin-based IR indices with renal outcomes and all-cause mortality, the included patients were stratified based on four quartiles of the TyG index. Informed consent for participation was obtained from all patients. This study was approved by the Institutional Review Board of Kaohsiung Medical University Hospital (approval number: KMUH-IRB-990198).

### 2.2. Collection of demographic, medical, and laboratory data

The baseline variables included the patients’ demographic characteristics, such as age, BMI, waist circumference (WC), and sex; medical history, such as cardiovascular disease, diabetes, hypertension, mean blood pressure (BP), antihypertensive drug use, Charlson comorbidity index, MetS, and malnutrition–inflammation–cachexia syndrome (MICS); and laboratory findings, such as eGFR, urine protein-to-creatinine ratio (Upcr), hemoglobin level, albumin level, C-reactive protein (CRP) level, total cholesterol level, and TG level. The demographic characteristics served as baseline variables. Data regarding patients’ medical history were obtained by reviewing their medical charts and interviewing them. The definitions of indicators were listed below. Biochemistry measurements were performed during screening and baseline visits and then every 3 months, as per the protocol. Laboratory data obtained from 3 months before the baseline to 3 months after it were averaged and analyzed (Table 1).

**Table 1:** Definitions of indicators.

Indicators	Definition
BMI (Body mass index)	Weight (in kg) divided by height squared (in m^2^).
WC (Waist circumference)	Performed in accordance with the protocol outlined by the World Health Organization (37).
TyG index	Ln [fasting TG level × fasting glucose level/2] (23).
TG/HDL-c ratio	Fasting TG level/HDL-c level (25).
TyG–BMI index	TyG index value × BMI (20).
METS-IR	Ln [(2 × fasting glucose level) + (fasting TG level) × BMI]/Ln (HDL-c level) (22).
MetS (Metabolic syndrome)	MetS components comprised a WC of ≥90 cm in men and ≥ 80 cm in women; systolic BP of ≥130 mmHg, diastolic BP of ≥85 mmHg, or hypertension; a HDL-c level of >40 mg/dl in men and >50 mg/dl in women; a TG level of ≥150 mg/dl; and a fasting blood glucose level of ≥100 mg/dl or a confirmed diagnosis of diabetes.
Charlson comorbidity index	Predicts mortality associated with the following 17 comorbidities: acute myocardial infarction, congestive heart failure, peripheral vascular disease, cerebral vascular accident, dementia, pulmonary disease, connective tissue disorder, peptic ulcer, liver disease, diabetes, diabetes complications, paraplegia, renal disease, cancer, metastatic cancer, severe liver disease, and human immunodeficiency virus infection (38).
Mean arterial pressure	Sum of one-third of the average systolic BP and two-thirds of the average diastolic BP, which were measured 3 months before and after patient enrollment.
Upcr (Urine protein-to-creatinine ratio)	Ratio of protein (in milligrams) and creatinine (in grams) in a random spot urine sample.

### 2.3. Outcomes

Renal outcomes of interest were renal replacement therapy and a 50% decrease in eGFR. All-cause mortality was ascertained by reviewing death certificates, patient charts, or the National Death Index. The models constructed to assess all-cause mortality included patients who had undergone renal replacement therapy; patients were censored only at death or the end of follow-up.

### 2.4. Statistical analysis

The baseline characteristics of all the patients were stratified using the TyG index. Categorical data were presented in terms of numbers and percentages. Continuous data with a normal distribution were presented in terms of mean ± standard deviation values, whereas those with a skewed distribution were presented as median and interquartile range values. Between-group differences were evaluated using a chi-squared test for categorical variables and a one-way analysis of variance for continuous variables. Cox proportional hazards regression analysis was performed to investigate the association of non-insulin-based IR indices with renal outcomes and all-cause mortality. Continuous variables with a skewed distribution were log-transformed to ensure a normal distribution. Covariates were selected after their clinical relevance was considered; this approach is consistent with that of our previous study (39). We adjusted for the effects of the following covariates: age, sex, eGFR, Upcr (log value), cardiovascular disease, smoking, cancer, severe liver disease, hypertension, hemoglobin level, BMI, cholesterol level (log value), glycosylated hemoglobin level, albumin level, CRP (ln value), and phosphorus level. All analyses were performed using SPSS for Windows (version 20.0; IBM, Chicago, IL, USA).

## 3. Results

### 3.1. Baseline characteristics of patients with stage 1–4 CKD stratified by TyG index

The patients (N = 2,457) were stratified by TyG index quartiles (Table 2). Of the patients, 35.8% were women, 22.0% had cardiovascular disease, 60.5% had hypertension, 49.3% had diabetes, and 66.5% had MetS. Their mean age was 62.6 ± 14.4 years, their mean eGFR was 40.5 ± 23.1 ml/min/1.73 m^2^, their mean BMI was 24.93 ± 3.61 kg/m^2^, and their median Upcr was 685 (246–1,804) mg/g. In patients with stage 1–4 CKD, BMI, WC, sex, diabetes, mean BP, antihypertensive drug use, MetS, Upcr, hemoglobin level, total cholesterol level, TG level, and all-cause mortality increased with an increase in TyG index. However, age, MICS, albumin level, and progression to ESRD decreased with an increase in TyG index.

**Table 2:** Baseline characteristics of patients with stage 1–4 chronic kidney disease stratified by triglyceride–glucose index.

	Triglyceride-glucose index				*p* value^#^
	Q1	Q2	Q3	Q4	
No. of patients (*n* = 2,457)	614 (25.0%)	614 (25.0%)	615 (25.0%)	614 (25.0%)	
Demographics/Medical history					
Age (years)	62.2 (16.3)	64.4 (14.2)	62.9 (14.0)	60.9 (12.7)	<0.001
Body mass index (kg/m^2^)	23.6 (3.5)	24.7 (3.4)	25.4 (3.4)	26.0 (3.6)	<0.001
Waist (cm)	84.3 (12.4)	87.6 (12.2)	89.6 (11.9)	91.2 (12.0)	<0.001
Sex (Female)	201 (32.7%)	213 (34.7%)	220 (35.8%)	245 (39.9%)	0.009
Cardiovascular disease	129 (21.0%)	149 (24.3%)	138 (22.4%)	125 (20.4%)	0.613
Diabetes mellitus	196 (31.9%)	248 (40.4%)	320 (52.0%)	447 (72.8%)	<0.001
Hypertension	341 (55.5%)	383 (62.4%)	388 (63.1%)	374 (60.9%)	0.056
Antihypertensive drug	238 (38.8%)	256 (41.7%)	283 (46.0%)	295 (48.0%)	<0.001
Mean BP (mmHg)	96.48 (12.85)	98.44 (13.07)	100.51 (13.57)	101.67 (13.54)	<0.001
Charlson score	3.27 (2.12)	3.38 (2.08)	3.36 (2.03)	3.36 (1.95)	0.792
Metabolic syndrome	226 (36.8%)	334 (54.4%)	509 (82.8%)	566 (92.2%)	<0.001
Malnutrition–inflammation	306 (49.8%)	279 (45.4%)	281 (45.7%)	268 (43.6%)	0.042
Laboratory data					
eGFR (ml/min/1.73 m^2^)	36.6 (25.0–50.4)	35.5 (24.5–49.3)	35.7 (25.6–48.4)	32.5 (23.4–46.5)	0.054
UPCR (mg/g)	448 (162–1,182)	570 (224–1,505)	771 (282–1754)	1,231 (369–3,131)	<0.001
Hemoglobin (g/dl)	12.0 (2.2)	12.1 (2.2)	12.4 (2.2)	12.3 (2.2)	0.020
Albumin (g/dl)	3.96 (0.47)	3.90 (0.54)	3.90 (0.56)	3.88 (0.59)	0.039
C-reactive protein (mg/L)	0.8 (0.3–4.9)	1.1 (0.3–3.9)	1.0 (0.3–4.5)	1.2 (0.4–5.0)	0.248
Total cholesterol (mg/dl)	178.9 (40.8)	195.5 (45.8)	205.8 (57.8)	225.9 (72.4)	<0.001
Triglyceride (mg/dl)	74.3 (23.7)	113.3 (21.8)	158.5 (40.0)	290.0 (251.6)	<0.001
Outcomes					
ESRD	108 (17.6%)	91 (14.8%)	93 (15.1%)	79 (12.9%)	<0.001
All-cause mortality	150 (24.4%)	163 (26.5%)	159 (25.9%)	232 (37.8%)	<0.001

Data are presented in terms of means (standard error), medians (interquartile range), or numbers (%). BP, blood pressure; eGFR, estimated glomerular filtration rate; Upcr, urine protein-to-creatinine ratio; CKD, chronic kidney disease; ESRD, end-stage renal disease.

# *p* value: Chi square test for categorical variable and one-way analysis of variance for continuous variable.

A multivariate linear regression model was constructed for the TyG index (Supplementary Table 1); the regression analysis results revealed significant (*p* < 0.05) increases in Upcr (log value), diabetes, WC, BMI, hemoglobin level, TG level, and albumin level with an increasing value in TyG index. By contrast, a significant (*p* < 0.05) decrease was noted in eGFR with an increase in TyG index.

### 3.2. Association between MetS and non-insulin-based IR indices

We determined the association between MetS prevalence and four IR indices by using a fully adjusted logistic regression model (Table 3). MetS prevalence significantly increased with an increase in TyG index. The odds ratio (OR) with 95% confidence interval (CI) values corresponding to TyG index quartiles Q2, Q3, and Q4 were 2.00 (1.50–2.67), 11.12 (7.91–15.65), and 28.19 (18.18–43.70), respectively, compared with the values corresponding to TyG index quartile Q1. Similar results with higher ORs were obtained for the other indices. MetS prevalence increased considerably with an increase in the values of these indices. The OR values corresponding to Q4 quartiles of the TG/HDL-c ratio, TyG–BMI index, and METS-IR were 48.22 (30.32–76.67), 29.77 (19.11–46.37), and 72.66 (43.67–120.90), respectively, compared with those corresponding to Q1. The *p* values for the aforementioned comparisons were <0.001.

**Table 3:** Hazard ratios corresponding to metabolic syndrome stratified by the quartiles of various insulin resistance indices.

	Q1	Q2	Q3	Q4	*p* for trend
Triglyceride-glucose index (TyG index)
Unadjusted	1 (reference)	2.05 (1.63–2.57)*	8.24 (6.32–10.75)*	20.24 (14.45–28.36)*	<0.001
Fully-adjusted	1 (reference)	2.00 (1.50–2.67)*	11.12 (7.91–15.65)*	28.19 (18.18–43.70)*	<0.001
Triglyceride/high density lipoprotein (TG/HDL-c ratio)
Unadjusted	1 (reference)	2.56 (2.03–3.22)*	8.73 (6.71–11.37)*	32.53 (22.24–47.56)*	<0.001
Fully-adjusted	1 (reference)	2.87 (2.13–3.88)*	15.55 (10.93–22.13)*	48.22 (30.32–76.67)*	<0.001
Triglyceride glucose-body mass index (TyG-BMI index)
Unadjusted	1 (reference)	3.37 (2.66–4.26)*	8.80 (6.77–11.43)*	26.33 (18.65–37.18)*	<0.001
Fully-adjusted	1 (reference)	3.81 (2.83–5.13)*	9.62 (6.89–13.44)*	29.77 (19.11–46.37)*	<0.001
Metabolic score for insulin resistance (METS-IR)
Unadjusted	1 (reference)	4.01 (3.16–5.10)*	12.26 (9.33–16.11)*	54.66 (35.97–83.05)*	<0.001
Fully-adjusted	1 (reference)	4.56 (3.35–6.19)*	16.64 (11.68–23.70)*	72.66 (43.67–120.90)*	<0.001

Data are presented in terms of hazard ratios and 95% confidence intervals. The fully adjusted model was adjusted for age, sex, estimated glomerular filtration rate, urine protein-to-creatinine ratio (log value), cardiovascular disease, smoking, cancer, severe liver disease, hypertension, hemoglobin level, body mass index, cholesterol level (log value), glycosylated hemoglobin level, albumin level, C-reactive protein level (ln value), and phosphorus level. TyG, triglyceride (TG)–glucose; BMI, body mass index; HDL-c, high-density lipoprotein cholesterol; METS-IR, metabolic score for insulin resistance.

* *p* < 0.001, compared with the reference TyG index, TG/HDL-c ratio, TyG–BMI index, or METS-IR.

### 3.3. Association of MetS with renal outcomes and all-cause mortality

Table 4 presents the hazard ratios (HRs) corresponding to renal outcomes and all-cause mortality stratified by MetS. In the fully adjusted Cox regression model adjusted for BMI and traditional confounding factors, patients with MetS had substantially high risks of adverse renal outcomes (HR: 1.56; 95% CI: 1.27–1.19). However, patients with MetS exhibited only marginal increases in the risk of all-cause mortality (HR: 1.17; 95% CI: 0.91–1.49; *p* = 0.216).

**Table 4:** Hazard ratios corresponding to renal outcomes and all-cause mortality stratified by metabolic syndrome.

	Metabolic syndrome		
	(−)	(+)	*p* for trend
HR for renal outcome
Unadjusted	1 (reference)	1.69 (1.44–1.99)*	< 0.001
Fully adjusted	1 (reference)	1.56 (1.27–1.91)*	< 0.001
HR for all-cause mortality	
Unadjusted	1 (reference)	1.57 (1.29–1.91)*	< 0.001
Fully adjusted	1 (reference)	1.17 (0.91–1.49)	0.216

Data are presented in terms of HRs and 95% confidence intervals. The fully adjusted model was adjusted for age, sex, estimated glomerular filtration rate, urine protein-to-creatinine ratio (log value), cardiovascular disease, smoking, cancer, severe liver disease, hypertension, hemoglobin level, body mass index, cholesterol level (log value), glycosylated hemoglobin level, albumin level, C-reactive protein level (ln value), and phosphorus level. HR, hazard ratio.

* *p* < 0.001, compared with patients with CKD without metabolic syndrome.

### 3.4. Association of non-insulin-based IR indices with renal outcomes and all-cause mortality

We investigated the association between adverse renal outcomes and non-insulin-based IR indices by using a fully adjusted Cox regression model (Table 5). A U-shaped association was identified between the TyG index and adverse renal outcomes. The risk of adverse renal outcomes markedly increased in Q1 (HR: 1.44; 95% CI: 1.13–1.84), Q2 (HR: 1.57; 95% CI: 1.26–1.95), and Q4 (HR: 1.38; 95% CI: 1.12–1.70) of the TyG index compared with that in the reference group (TyG index Q3). A reverse association was found between the TyG–BMI index and adverse renal outcomes. The risk of adverse renal outcomes was significantly higher in Q1 (HR: 1.86; 95% CI: 1.19–2.91) and Q2 (HR: 1.57; 95% CI: 1.10–2.23) of the TyG–BMI index compared with that in the reference group (TyG–BMI index Q4). No prominent associations were observed between TG/HDL-c ratio, METS-IR, and adverse renal outcomes.

**Table 5:** Hazard ratios corresponding to renal outcome stratified by the quartiles of various insulin resistance indices.

	Q1	Q2	Q3	Q4	*p* for trend
Triglyceride-glucose index (TyG index)
Unadjusted	0.89 (0.72–1.11)	1.03 (0.84–1.27)	1 (reference)	1.61 (1.33–1.95)*	< 0.001
Fully-adjusted	1.44 (1.13–1.84)*	1.57 (1.26–1.95)*	1 (reference)	1.38 (1.12–1.70)*	< 0.001
Triglyceride/high density lipoprotein (TG/HDL-c ratio)
Unadjusted	1.03 (0.83–1.28)	1 (reference)	1.26 (1.02–1.54)*	1.53 (1.26–1.87)*	< 0.001
Fully-adjusted	1.17 (0.93–1.47)	1 (reference)	1.00 (0.81–1.25)	1.06 (0.85–1.31)	0.518
Triglyceride glucose-body mass index (TyG-BMI index)
Unadjusted	0.94 (0.77–1.14)	0.82 (0.67–1.00)	0.87 (0.71–1.06)	1 (reference)	0.223
Fully-adjusted	1.86 (1.19–2.91)*	1.57 (1.10–2.23)*	1.17 (0.90–1.53)	1 (reference)	0.033
Metabolic score for insulin resistance (METS-IR)
Unadjusted	1.00 (0.81–1.22)	0.95 (0.77–1.16)	1 (reference)	1.16 (0.96–1.41)	0.196
Fully-adjusted	1.29 (0.93–1.79)	1.06 (0.84–1.33)	1 (reference)	1.07 (0.85–1.34)	0.432

Data are presented in terms of hazard ratios and 95% confidence intervals. The fully adjusted model was adjusted for age, sex, estimated glomerular filtration rate, urine protein-to-creatinine ratio (log value), cardiovascular disease, smoking, cancer, severe liver disease, hypertension, hemoglobin level, body mass index, cholesterol level (log value), glycosylated hemoglobin level, albumin level, C-reactive protein level (ln value), and phosphorus level. TyG, triglyceride (TG)–glucose; BMI, body mass index; HDL-c, high-density lipoprotein cholesterol; METS-IR, metabolic score for insulin resistance.

* *p* < 0.001, compared with the reference TyG index, TG/HDL-c ratio, TyG–BMI index, or METS-IR.

We also investigated the association between all-cause mortality and non-insulin-based IR indices by using a fully adjusted Cox regression model (Table 6). The TyG and TyG–BMI indices were reversely associated with all-cause mortality. The risk of all-cause mortality was significantly higher in Q1 of the TyG index (HR: 1.38; 95% CI: 1.08–1.76) compared with that in the reference group (TyG index Q2). Furthermore, this risk was significantly higher in the Q1 quartile of the TyG–BMI index (HR: 1.87; 95% CI: 1.11–3.14) compared with that in the reference group (TyG–BMI index Q4). No strong associations were found between all-cause mortality and TG/HDL-c ratio or METS-IR.

**Table 6:** Hazard ratios corresponding to all-cause mortality stratified by the quartiles of various insulin resistance indices.

	Q1	Q2	Q3	Q4	*p* for trend
Triglyceride-glucose index (TyG index)
Unadjusted	1.04 (0.82–1.32)	1 (reference)	0.97 (0.76–1.24)	0.94 (0.73–1.20)	0.855
Fully-adjusted	1.38 (1.08–1.76)*	1 (reference)	1.17 (0.91–1.50)	1.03 (0.77–1.37)	0.066
Triglyceride/high density lipoprotein (TG/HDL-c ratio)
Unadjusted	1.02 (0.80–1.31)	1.18 (0.93–1.50)	1.03 (0.80–1.32)	1 (reference)	0.506
Fully-adjusted	1.17 (0.89–1.53)	1.23 (0.96–1.59)	1.00 (0.78–1.30)	1 (reference)	0.256
Triglyceride glucose-body mass index (TyG-BMI index)
Unadjusted	1.53 (1.21–1.95)*	1.11 (0.86–1.44)	1.14 (0.88–1.47)	1 (reference)	0.003
Fully-adjusted	1.87 (1.11–3.14)*	1.24 (0.82–1.89)	1.07 (0.77–1.49)	1 (reference)	0.049
Metabolic score for insulin resistance (METS-IR)
Unadjusted	1.15 (0.90–1.47)	1.14 (0.89–1.46)	1 (reference)	1.08 (0.85–1.38)	0.670
Fully-adjusted	1.17 (0.82–1.66)	1.11 (0.85–1.45)	1 (reference)	1.17 (0.88–1.56)	0.654

Data are presented in terms of hazard ratios and 95% confidence intervals. The fully adjusted model was adjusted for age, sex, estimated glomerular filtration rate, urine protein-to-creatinine ratio (log value), cardiovascular disease, smoking status, cancer, severe liver disease, hypertension, hemoglobin level, body mass index, cholesterol level (log value), glycosylated hemoglobin level, albumin level, C-reactive protein level (ln value), and phosphorus level. TyG, triglyceride (TG)–glucose; BMI, body mass index; HDL-c, high-density lipoprotein cholesterol; METS-IR, metabolic score for insulin resistance.

* *p* < 0.001, compared with the reference TyG index, TG/HDL-c ratio, TyG–BMI index, or METS-IR.

### 3.5. Association of BMI with renal outcomes and mortality in patients with stage 1–4 CKD

We stratified the HRs corresponding to renal outcomes and all-cause mortality by BMI (Supplementary Table 2). A high BMI was associated with adverse renal outcomes, whereas a low BMI was associated with a higher risk of all-cause mortality. Compared with patients with a BMI of 25.1–27.5 kg/m^2^ (reference group), the risk of adverse renal outcomes was significantly higher in those with a BMI of 27.6–30.0 kg/m^2^ (HR: 1.31; 95% CI: 1.02–1.69) and those with a BMI of 30.1–35.0 kg/m^2^ (HR: 1.48; 95% CI: 1.12–1.94). Compared with patients with a BMI of 27.6–30.0 kg/m^2^ (reference group), the risk of all-cause mortality was significantly higher in those with a BMI of 15.1–20.0 kg/m^2^ (HR: 1.71; 95%: 1.15–2.54) and marginally higher in those with a BMI of 20.1–22.5 kg/m^2^ (HR: 1.39; 95% CI: 0.99–1.96).

### 3.6. HRs corresponding to renal outcomes and all-cause mortality stratified by fasting TG and glucose levels

We analyzed the association of fasting TG and glucose levels with renal outcomes and all-cause mortality by using a Cox regression model (Supplementary Table 3). Low, but not high, fasting TG levels were associated with adverse renal outcomes and a heightened risk of all-cause mortality. A higher risk of all-cause mortality was observed in patients with fasting TG levels of <50 mg/dl (HR: 1.41; 95% CI: 0.83–2.38) and 50–100 mg/dl (HR: 1.34; 95% CI: 1.01–1.78) compared with that in patients with a fasting TG level of 150–200 mg/dl (reference group). Patients with a fasting TG level of <50 mg/dl had a marginally higher risk of all-cause mortality (HR: 1.35; 95% CI: 0.84–2.17).

High fasting glucose levels were associated with adverse renal outcomes and a heightened risk of all-cause mortality. Patients with a fasting glucose level of >150 mg/dl had elevated risks of adverse renal outcomes (HR: 1.32; 95% CI: 1.05–1.65) and all-cause mortality (HR: 1.43; 95% CI: 1.09–1.88). Low fasting glucose levels were not associated with these adverse clinical outcomes.

## 4. Discussion

In the present study, four non-insulin-based IR indices (TyG index, TyG–BMI index, TG/HDL-c ratio, and METS-IR) were found to be associated with MetS. Compared with patients without MetS, patients with MetS exhibited a significantly higher risk of adverse renal outcomes and a marginally higher risk of all-cause mortality. The current evidence between insulin-based IR indices and clinical outcomes were debatable and few studies have explored the association between non-insulin-based IR indices with mortality and renal outcomes. Notably, low values of the TyG and TyG–BMI indices were paradoxically associated with higher risks of adverse renal outcomes and all-cause mortality (Tables 5, 6). Furthermore, among the IR indices, only high TyG index was associated with adverse renal outcomes. This paradox may be explained on the basis of the associations of low BMI and low fasting TG level with poor clinical outcomes, considering the effects of their components. Early screening by MetS or TyG index could help to predict clinical outcomes in patients with stage 1–4 CKD.

Our results support the value of non-insulin-based IR indices for predicting MetS in patients with CKD. The TyG index appears to be a better biomarker of MetS than HOMA-IR (40) and IR indices have distinct power for MetS detection (27, 41). In the present study, four non-insulin-based IR indices (TyG index, TyG–BMI index, TG/HDL-c ratio, and METS-IR) effectively predicted MetS in patients with CKD. The Q4 quartile of the METS-IR index had the highest OR (72.66; 95% CI: 43.67–120.90). Although we considered the obesity paradox in our study, MetS was found to be associated with a significantly higher risk of adverse renal outcomes and a marginally higher risk of all-cause mortality. These findings corroborate the effects of BMI observed in our cohort (mean BMI: 24.93 ± 3.61 kg/m^2^). A high BMI was identified as a risk factor for adverse renal outcomes but not all-cause mortality. These findings are consistent with those of Navaneethan et al., who conducted a relevant study involving 25,868 patients with stage 3 or 4 CKD (8). The African American Study of Kidney Disease and Hypertension reported no association of MetS with CKD progression or mortality in patients (*N* = 842) with CKD and hypertension (mean BMI: 31.4 ± 7.0 kg/m^2^; eGFR: 25–65 ml/min/1.73 m^2^) (7). However, Pammer et al. revealed significant increases in the risks of all-cause mortality and cardiovascular events with an increased prevalence of MetS in a large cohort comprising German patients (*N* = 5,110) with stage 1–3 CKD (mean BMI: 29.8 ± 6.0 kg/m^2^) (42). These differences may be explained on the basis of the distribution of patients with a low BMI or malnutrition. Nonetheless, MetS remains a major risk factor for CKD progression.

The effects of MetS on renal outcomes may be explained on the basis of IR. IR is associated with metabolite-driven gluconeogenesis and ectopic lipid accumulation (14), which is associated with the glucose–fatty acid cycle (43). In patients with IR, the pathway-specific impairment of phosphatidylinositol 3-kinase–dependent signaling may result in an imbalance between the production of nitric oxide and the secretion of endothelin-1, which leads to endothelial dysfunction (44). IR also promotes the development of cardiovascular diseases by inducing oxidative stress and eliciting inflammatory responses (45), further leading to endothelial dysfunction and atherosclerotic plaque formation (46). Obesity causes a glomerular disease called obesity-related glomerulopathy (47), which increases the incidence of CKD (48) and ESRD (49). The mechanisms underlying obesity-related renal injury involve hemodynamic changes, inflammation, oxidative stress, apoptosis, and renal scarring (50). IR and impaired glucose tolerance result in obesity (51) and proteinuria (52). MetS leads to increased incidences of CKD (4). The ability of non-insulin-based IR indices to predict renal outcomes in patients with CKD remains to be confirmed. A large-scale Austrian study revealed a strong association between the TyG index and ESRD risk (29). Shang et al. demonstrated a U-shaped association between the TyG index and diabetic nephropathy (31). In the present study, adverse renal outcomes exhibited a U-shaped association with the TyG index and a reverse association with the TyG–BMI index. This U-shaped association may be explained on the basis of fasting TG level and BMI.

The association between insulin-based IR indices and clinical outcomes remains debatable. The findings of the studies on the association between HOMA-IR and clinical outcomes have been inconsistent. A study involving 1,883 patients with mild to moderate CKD without diabetics revealed no associations of HOMA-IR with renal outcomes, cardiovascular events, or all-cause mortality (53). Furthermore, a study involving patients with stage 2–4 CKD reported no apparent differences between patients with IR and those without IR (assessed using HOMA-IR) in terms of eGFR (54). CKD progression was slower in patients with lower HOMA-IR; however, this insulin-based index failed to predict cardiovascular events or all-cause mortality (55). Nevertheless, HOMA-IR was reported to be an independent predictor of cardiovascular mortality in patients with ESRD (56). A positive association was identified between this insulin-based index and CKD progression in Korean adults without diabetes (57). Data regarding IR indices in patients with CKD remain limited; hence, further studies are required on insulin-based and non-insulin-based IR (particularly TyG index) indices to reveal their diagnostic value for patients with CKD.

Among the non-insulin-based IR indices, low values of TyG and TyG–BMI indices were associated with risk of adverse clinical outcomes; meanwhile, only high TyG index was associated with adverse renal outcomes. Few studies have explored the associations between non-insulin-based IR indices and all-cause mortality. A U-shaped association was observed between the TyG index and all-cause mortality in the general population (32). This index is also associated with major cardiovascular events in patients with CKD with (58) and without (59) diabetes. Our findings revealed reverse associations between the TyG and TyG–BMI indices and all-cause mortality in patients with CKD. This paradox may be explained on the basis of the associations of low fasting TG level and low BMI with the risk of all-cause mortality in these patients. Thus, another obesity paradox is evident from the fact that low TyG and TyG–BMI indices are indicators of poor clinical outcomes.

BMI is strongly associated with mortality in the general population; the association had a J-shaped curve in a study of 1.46 million White adults (60) and a U-shaped curve in a study of 0.85 million East Asian adults (61). Vascular diseases, such as ischemic heart disease and stroke, contribute to mortality in patients with obesity (62). The obesity paradox (63), which is characterized by a high BMI and a low mortality risk, is observed in patients with CKD in whom protein-energy wasting (PEW) and inflammation serve as effective predictors of an early death (64). In these patients, malnutrition–inflammation–cachexia syndrome (MICS) was common and was identified to be the main cause of cardiovascular disease (65) and mortality (66). CKD and ESRD have been reported to be associated with PEW (67) and MICS (66). A low BMI ensures an increased risk of mortality (68). We previously reported a U-shaped association of waist-to-hip ratio with all-cause mortality and a reverse association of BMI with adverse renal outcomes and all-cause mortality in patients with CKD (10, 35). Therefore, MICS, PEW, and inflammation may strongly affect the reverse association between IR indices and adverse clinical outcomes.

Non-insulin-based IR indices are affected by their components. We investigated the associations of the two components of TyG index, namely fasting TG and glucose levels, with clinical outcomes. The results suggested that low fasting TG level and high fasting glucose level were associated with poor clinical outcomes. In patients with CKD presenting with MICS, a lower TG level has been associated with a higher risk of all-cause mortality (69). An elevated glucose level is a risk factor for adverse macrovascular and microvascular outcomes (70), which in turn increase the risks of all-cause mortality and adverse renal outcomes. The predictive value of the TyG index for adverse renal outcomes may be explained on the basis of the strong effects of fasting glucose level over fasting TG level.

The primary strength of the present study lies in its large sample size (*N* = 2,457) and inclusion of patients with stage 1–4 CKD and a BMI of 15.0–35.0 kg/m^2^. To our knowledge, this is the first study to explore the associations of various non-insulin-based IR indices with adverse renal outcomes and all-cause mortality in patients with CKD. The present study had some limitations. First, the cohort comprised East Asian patients; therefore, the effects of ethnicity on body composition and clinical outcomes could not be investigated in this study. Second, baseline anthropometric measurements were used in the regression analysis without considering the possible time-dependent changes in the variables. Third, dietary and medication factors were not included in the Cox regression models; these factors influence obesity and CKD. Fourth, we included patients with only stage 1–4 CKD and not those with stage 5 CKD; therefore, our findings may not be applicable to all patients with CKD. Future studies are warranted to clarify the nature of the IR index paradox and explore the efficacies of various IR indices for predicting adverse renal outcomes and all-cause mortality.

## 5. Conclusion

In the present study, which involved patients with stage 1–4 CKD, high non-insulin-based IR indices were associated with MetS. Patients with MetS exhibited elevated risks of adverse renal outcomes and all-cause mortality. Among the four non-insulin-based indices assessed in this study, only a high TyG index was associated with adverse renal outcomes, whereas low TyG and TyG–BMI indices were associated with all adverse clinical outcomes. The obesity paradox may alter the predictive value of these indices. Early screening by MetS or TyG index could help to predict clinical outcomes in patients with stage 1–4 CKD. In the future, large-scale studies should focus on comparing insulin-based and non-insulin-based IR indices to determine their relative predictive values. Our findings may facilitate the early screening of renal outcomes and other clinical outcomes in patients with stage 1–4 CKD. This study may serve as a reference for relevant future studies.

## Data availability statement

The raw data supporting the conclusions of this article will be made available by the authors, without undue reservation.

## Ethics statement

The studies involving human participants were reviewed and approved by Institutional Review Board of Kaohsiung Medical University Hospital. The patients/participants provided their written informed consent to participate in this study.

## Author contributions

F-CS, S-WN, and C-CH: conceptualization, formal analysis, methodology, and writing—original draft. J-MC: supervision. HY-HL, W-CT, I-CK, Y-KC, and Y-LC: writing—review and editing. All authors have read and agreed to the published version of the manuscript. All authors contributed to the article and approved the submitted version.

## Conflict of interest

The authors declare that the research was conducted in the absence of any commercial or financial relationships that could be construed as a potential conflict of interest.

## Publisher’s note

All claims expressed in this article are solely those of the authors and do not necessarily represent those of their affiliated organizations, or those of the publisher, the editors and the reviewers. Any product that may be evaluated in this article, or claim that may be made by its manufacturer, is not guaranteed or endorsed by the publisher.
