# Supplementary material for: Non-insulin-based insulin resistance indices for predicting all-cause mortality and renal outcomes in patients with stage 1–4 chronic kidney disease: another paradox
Source: Front Nutr. 2023 May 15;10:1136284. doi: 10.3389/fnut.2023.1136284 (PMC10225593; doi:10.3389/fnut.2023.1136284)
Supplement: Supplementary file 1 [file Data_Sheet_1.docx]

Supplementary Material

**Supplementary Table 1 | Results of multivariate linear regression that included the triglyceride–glucose index**

|  | Beta coefficient | 95% CI Beta coefficient | *P* value |
| --- | --- | --- | --- |
| Constant | 4.455 |  |  |
| Age (years) | 0.000 | -0.002 to 0.001 | 0.517 |
| Gender (male) | -0.038 | -0.080 to 0.004 | 0.074 |
| eGFR (ml/min/1.73 m^2^) | -0.001 | -0.002 to 0.000 | 0.035 |
| Upcrlog | 0.076 | 0.039 to 0.112 | <0.001 |
| Diabetes | 0.183 | 0.139 to 0.228 | <0.001 |
| Waist (cm) | 0.045 | 0.003 to 0.087 | 0.037 |
| BMI (Kg/m^2^) | 0.011 | 0.005 to 0.017 | <0.001 |
| Hemoglobin (g/dl) | 0.011 | 0.000 to 0.022 | 0.041 |
| Triglyceride log | 0.804 | 0.764 to 0.844 | <0.001 |
| Albumin (g/dl) | 0.078 | 0.039 to 0.118 | <0.001 |
| CRP ln | 0.001 | -0.018 to 0.021 | 0.903 |

Abbreviations: CI, confidence interval; eGFR, estimated glomerular filtration rate; Upcr, urine protein-to-creatinine ratio; BMI, body mass index; and CRP, C-reactive protein.

**Supplementary Table 2 | Hazard ratios corresponding to renal outcomes and all-cause mortality stratified by body mass index**

|  | **Body mass index (kg/m^2^)** | | | | | |
| --- | --- | --- | --- | --- | --- | --- |
|  | **G1** | **G2** | **G3** | **G4** | **G5** | **G6** |
|  | **15.0-20.0** | **20.1-22.5** | **22.6-25.0** | **25.1-27.5** | **27.6-30.0** | **30.1-35.0** |
| **HR for renal outcome** | | | | | | |
| Unadjusted | 1.21 (0.91-1.61) | 1.35 (1.08-1.69)* | 1.11 (0.91-1.36) | 1 (reference) | 1.10 (0.86-1.41) | 1.26 (0.96-1.64) |
| Fully adjusted | 1.02 (0.75-1.39) | 1.19 (0.94-1.50) | 1.15 (0.93-1.42) | 1 (reference) | 1.31 (1.02-1.69)* | 1.48 (1.12-1.94)* |
| **HR for all-cause mortality** | | | | | | |
| Unadjusted | 1.83 (1.27-2.63)* | 1.39 (1.00-1.93) | 1.27 (0.94-1.73) | 1.20 (0.88-1.64) | 1 (reference) | 1.08 (0.73-1.61) |
| Fully adjusted | 1.71 (1.15-2.54)* | 1.39 (0.99-1.96) | 1.14 (0.83-1.55) | 1.20 (0.87-1.64) | 1 (reference) | 1.17 (0.78-1.75) |

Data are presented in terms of HRs and 95% confidence intervals.
The fully adjusted model was adjusted for age, sex, estimated glomerular filtration rate, urine protein-to-creatinine ratio (log value), cardiovascular disease, smoking status, cancer, severe liver disease, hypertension, hemoglobin level, body mass index, cholesterol level (log value), glycosylated hemoglobin level, albumin level, C-reactive protein level (ln value), and phosphorus level.
*p < .05, compared with the reference body mass index.
Abbreviation: HR, hazard ratio.

**Supplementary Table 3 |** Hazard ratios corresponding to renal outcomes and all-cause mortality stratified by fasting triglyceride and glucose levels

|  | **Fasting triglyceride (mg/dL)** | | | | | |
| --- | --- | --- | --- | --- | --- | --- |
|  | **G1**  **<50** | **G2**  **50-100** | **G3**  **100-150** | **G4**  **150-200** | **G5**  **200-250** | **G6**  **>250** |
| **HR for renal outcome** | | | | | | |
| Unadjusted | 0.79 (0.51-1.24) | 0.80 (0.63-1.00) | 0.94 (0.76-1.17) | 1 (reference) | 1.25 (0.96-1.64) | 1.37 (1.07-1.77)* |
| Fully adjusted | 1.35 (0.84-2.17) | 1.08 (0.84-1.37) | 1.00 (0.80-1.25) | 1 (reference) | 1.15 (0.88-1.52) | 1.13 (0.87-1.48) |
| **HR for all-cause mortality** | | | | | | |
| Unadjusted | 1.15 (0.70-1.90) | 1.27 (0.97-1.66) | 1.11 (0.85-1.45) | 1 (reference) | 1.02 (0.71-1.46) | 0.86 (0.60-1.22) |
| Fully adjusted | 1.41 (0.83-2.38) | 1.34 (1.01-1.78)* | 1.04 (0.79-1.37) | 1 (reference) | 1.06 (0.74-1.53) | 0.86 (0.59-1.25) |
|  | **Fasting glucose (mg/dL)** | | | | | |
|  | **G1**  **<99** | **G2**  **99-126** | **G3**  **126-150** | **G4**  **>150** | — | — |
| **HR for renal outcome** | | | | | | |
| Unadjusted | 1 (reference) | 1.00 (0.84-1.19) | 1.33 (1.05-1.68)* | 1.72 (1.41-2.09)** | — | — |
| Fully adjusted | 1 (reference) | 1.15 (0.96-1.38) | 1.06 (0.82-1.36) | 1.32 (1.05-1.65)* | — | — |
| **HR for all-cause mortality** | | | | | | |
| Unadjusted | 1.02 (0.83-1.26) | 1 (reference) | 1.30 (0.96-1.74) | 1.56 (1.22-2.00)** | — | — |
| Fully adjusted | 1.09 (0.87-1.35) | 1 (reference) | 1.14 (0.84-1.56) | 1.43 (1.09-1.88)* | — | — |

Data are presented in terms of HRs and 95% confidence intervals.
The fully adjusted model was adjusted for age, sex, estimated glomerular filtration rate, urine protein-to-creatinine ratio (log value), cardiovascular disease, smoking status, cancer, severe liver disease, hypertension, hemoglobin level, body mass index, cholesterol level (log value), glycosylated hemoglobin level, albumin level, C-reactive protein level (ln value), and phosphorus level.
*p < .05, compared with fasting triglyceride or glucose level.
**p < .01, compared with fasting glucose level.
Abbreviation: HR, hazard ratio.
